# Evaluation of deep vein thrombosis in patients with severe traumatic brain injuries

**DOI:** 10.1186/2197-425X-3-S1-A438

**Published:** 2015-10-01

**Authors:** P Mendez, A Chavez, L Avila, A Seifi

**Affiliations:** University of Texas Health Science Center at San Antonio, Neurosurgery, San Antonio, TX USA

## Introduction

Deep venous thrombosis (DVT), often prodromal for pulmonary embolism, is known to cause significant morbidity and mortality.^1^ The use of postoperative chemoprophylaxis is controversial among clinicians who care for severe traumatic brain injury patients due to concern of intracranial hemorrhage (ICH) and its pernicious effects.

## Objectives

To evaluate the incidence of DVT in severe traumatic brain injury (TBI), as well as the impact of chemoprophylaxis on development of DVT.

## Methods

We conducted a retrospective case control study with patients admitted to University Hospital neurosurgical ICU in San Antonio, Texas from 2011-2013. Severe TBI was defined as patients who required intracranial pressure monitoring within 48 hours of admission. Patients less than 18 years of age, DVT on admission, pregnancy, chronic anticoagulation, and death within 72 hours after TBI were excluded. Demographic data, etiology of TBI, complications, hospital length of stay (LOS), start date of chemoprophylaxis were gathered. Progression of ICH was defined as lesion expansion or development of a new ICH on a repeat CT scan. Fisher's exact test was used to determine incidence and mortality of DVT with and without chemoprophylaxis. The Mann-Whitney U test was used to determine hospital LOS.

## Results

Out of 396 qualifying records, 155 records entered the study group after exclusion criteria. The cohort was mostly composed of white (71.6%), male (76.8%) with a median age of 41. The majority types of TBIs were subdural hemorrhage (62.6%) & subarachnoid hemorrhage (60%). A total of 122 patients received chemoprophylaxis, the average number of days post admission to begin prophylaxis was 5.04 ± 3.95. The mean number of days post stable head CT being 6.69. Meaning some patients received chemoprophylaxis prior to stable CT. The incidence of DVT was 12.26% and PE 2.58%. We found 30.3% of patients who did not receive chemoprophylaxis developed a DVT vs. 7.38% of patients who did receive chemoprophylaxis. We observed 9.35 days longer LOS in those who developed a DVT, and did not receive chemoprophylaxis. Our study mortality rate was 18%. The incidence of ICH progression after chemoprophylaxis was 7.74%.

## Conclusions

Our data suggests a lower incidence of DVT in patients who received chemoprophylaxis and longer hospital LOS than the group who did not. We found improved mortality in patients who received chemoprophylaxis at any point of hospital stay. Thus, consider starting chemoprophylaxis to reduce complications of DVT, LOS and hospital costs. Additional larger prospective studies should confirm the best time to begin chemoprophylaxis in severe TBI patients.

## Grant Acknowledgment

UTHSCSA Neurosurgery, San Antonio, TX
